# Alchemical analysis of FDA approved drugs†

**DOI:** 10.1039/d3dd00039g

**Published:** 2023-08-30

**Authors:** Markus Orsi, Daniel Probst, Philippe Schwaller, Jean-Louis Reymond

**Affiliations:** a Department of Chemistry, Biochemistry and Pharmaceutical Sciences, University of Bern Freiestrasse 3 3012 Bern Switzerland jean-louis.reymond@unibe.ch; b Ecole Polytechnique Fédérale de Lausanne 1015 Lausanne Switzerland

## Abstract

Chemical space maps help visualize similarities within molecular sets. However, there are many different molecular similarity measures resulting in a confusing number of possible comparisons. To overcome this limitation, we exploit the fact that tools designed for reaction informatics also work for alchemical processes that do not obey Lavoisier's principle, such as the transmutation of lead into gold. We start by using the differential reaction fingerprint (DRFP) to create tree-maps (TMAPs) representing the chemical space of pairs of drugs selected as being similar according to various molecular fingerprints. We then use the Transformer-based RXNMapper model to understand structural relationships between drugs, and its confidence score to distinguish between pairs related by chemically feasible transformations and pairs related by alchemical transmutations. This analysis reveals a diversity of structural similarity relationships that are otherwise difficult to analyze simultaneously. We exemplify this approach by visualizing FDA-approved drugs, EGFR inhibitors, and polymyxin B analogs.

## Abstract 2

Reaction informatics is used to map the chemical space of drugs paired by similarity according to different molecular fingerprints.

## Graphical abstract

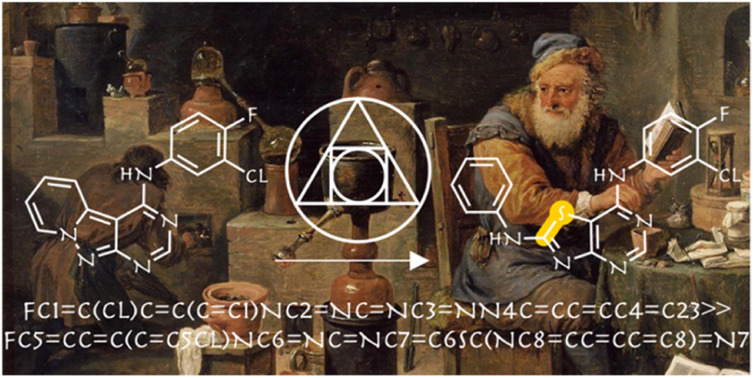

## Introduction

Mapping molecular databases in a chemical space where distances represent similarities between molecules helps to understand their structural similarities and identify relationships that can provide critical insights for drug development and related fields.^1–15^ However, molecular similarity can be computed in multiple ways,^16,17^ typically using various molecular fingerprints,^18^ resulting in a confusing multiplicity of possible chemical space representations.^19,20^

**Fig. 1:**
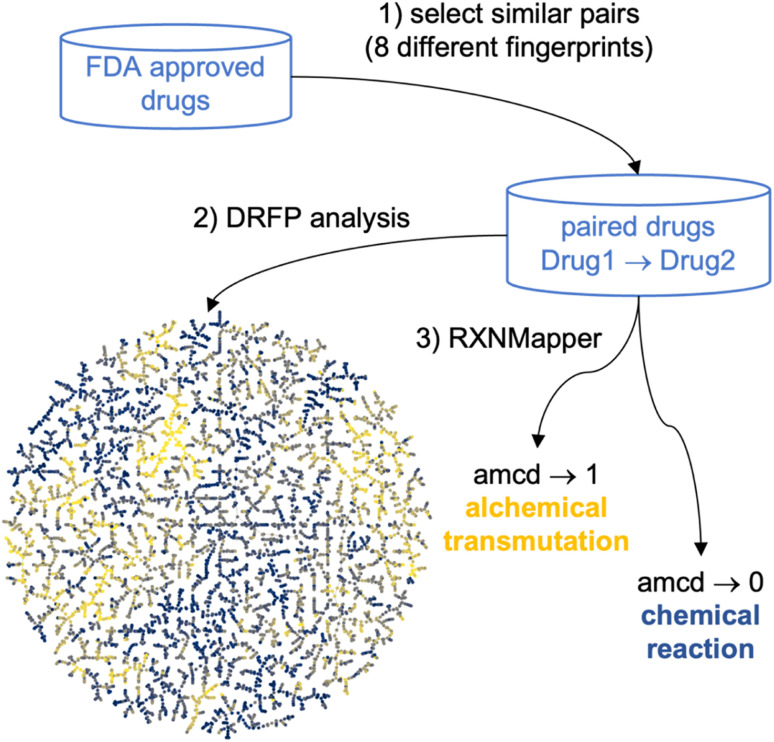
Principle of alchemical analysis of molecular sets at the example of FDA approved drugs. (1) Drugs pairs passing a similarity threshold according to eight different molecular fingerprints are selected. (2) The set of selected pairs is mapped in a TMAP computed using the differential reaction fingerprint (DRFP), color coded by the RXNmapper confidence distance (amcd). (3) The amcd distinguishes pairs of drugs related by a possible reaction (amcd → 0) from those related by an alchemical transmutation (amcd → 1).

To overcome this limitation and create a chemical space map considering various similarity measures simultaneously, we report a new approach of applying reaction informatics tools to map and analyze drug pairs, namely the differential reaction fingerprint (DRFP)^21^ and the Transformer-based RXNMapper model,^22–24^ respectively (Fig. 1). These tools were initially designed to analyze chemical reactions. However, they can also be applied to processes that do not obey Lavoisier's principle, the conservation of mass, such as the alchemical transmutation of lead into gold.^25,26^ Here, we apply them to transmutations between pairs of molecules selected for their similarity according to various molecular fingerprints as similarity measures, an approach related to the recent development of transformer models for drug optimization.^27,28^

We start by using DRFP, which encodes chemical reactions by storing the symmetric difference of two sets containing the circular molecular *n*-grams generated from the molecules of the molecular pair as a binary fingerprint,^21^ to represent the chemical space of drug pairs as a TMAP (tree-map).^29^ A TMAP lays out the minimum spanning tree of the nearest neighbor graphs according to a selected similarity measure, here DRFP, and represents a remarkably efficient dimensionality reduction method for high-dimensional datasets. The DRFP TMAP visualization provides a global similarity perspective across drug pairs combining the selected similarity measures. We then use RXNMapper,^22^ a model trained on one million reactions documented in the USPTO dataset^30^ to pair corresponding atoms between reactants and products in a chemical reaction, to identify the structural relationship between drugs. The confidence score of this transformer appears not to correlate with any of the molecular similarity measures used. It allows us to distinguish drug pairs related by feasible chemical processes, such as matched molecular pairs corresponding to substituent exchanges,^31,32^ from those related by more esoteric, alchemical transmutations including scaffold-hopping changes.^33,34^ We demonstrate this approach with the example of FDA-approved drugs as a diversity set, as well as for a series of EGFR inhibitors and polymyxin B analogs as two high similarity sets chosen among small molecule drugs and peptide macrocyclic drugs, respectively.

## Methods

### Datasets

The set of FDA-approved drugs was downloaded from ZINC15,^35,36^ the SMILES were canonicalized and kekulized and duplicates were removed to obtain a set of 1213 unique chemical structures. For the EGFR set, all compounds binding to the tyrosine kinase erbB1 with a molecular weight <700 and an annotated IC_50_ value were downloaded from ChEMBL-31.^37^ After SMILES canonicalization and kekulization, duplicates were removed and the 1500 molecules with the highest ECFP4 Tanimoto similarity to afatinib were selected for the final set. The polymyxin B similarity set was downloaded from ChEMBL-31 by selecting compounds above the 55% ChEMBL similarity threshold with annotated MIC values. The SMILES were canonicalized and kekulized, and duplicates were removed, resulting in a final set of 274 structures.

### Molecular fingerprints and similarity calculations

Chemical structures were encoded as eight different fingerprints, namely extended connectivity fingerprints ECFP4 and ECFP6,^38,39^ the MinHashed Fingerprint MHFP6,^40^ the RDKit Atom-Pair Fingerprint (AP),^41^ the Macromolecule Extended Fingerprint (MXFP),^42^ the MinHashed Atom-Pair fingerprint MAP4,^43^ the Molecular ACCess System keys (MACCS),^44^ and Molecular Quantum Numbers (MQNs).^45^ ECFP4, ECFP6, AP, MACCS and MQN were calculated using the implementation in the RDKit package (2022.3.4., https://www.rdkit.org). ECFPs were calculated as 2048-bit vectors. MHFP6 and MAP4 were calculated as 2048-bit vectors using the code described in https://github.com/reymond-group/mhfp and https://github.com/reymond-group/map4. MXFP was calculated using a new open-source version available at https://github.com/reymond-group/mxfp_python. The differential reaction fingerprint (DRFP)^21^ was calculated as 2048-bit vectors using the code available at https://github.com/reymond-group/drfp.

Pairwise distances for every possible molecular pair were calculated and stored as a matrix for each fingerprint. Distances were calculated as Jaccard distances (*d*_J_) for ECFP4, ECFP6, MHFP6, AP, MAP4 and MACCS keys, and as Taxicab distances (*d*_T_) for MXFP and MQNs, with values min–max standardized. We selected similar pairs by applying the following distance threshold: *d*_J_ < 0.6 for ECFP4, ECFP6, MHFP6, *d*_J_ < 0.5 for AP, *d*_J_ < 0.2 for MACCS, *d*_J_ < 0.8 for MAP4, *d*_T_ < 0.1 for MXFP and *d*_T_ < 0.05 for MQN (Taxicab distances after rescaling) for the FDA set and *d*_J_ < 0.2 for ECFP4, ECFP6, MHFP6, AP, *d*_J_ < 0.0125 for MACCS, *d*_J_ < 0.3 for MAP4, *d*_T_ < 0.1 for MXFP and *d*_T_ < 0.05 for MQN for the EGFR and PMB sets.

Additionally, the ranking of molecular pairs for every compound and fingerprint was calculated, resulting in 1213 ranked lists of 1213 pairs each for the FDA set, 1500 ranked lists of 1500 ranked pairs for the EGFR set and 274 ranked lists of 274 pairs for the polymyxin B similarity set for each fingerprint.

Violin plots to display the distribution of distances for every fingerprint and heatmaps to visualize correlations between fingerprints were generated using the seaborn (0.11.2) package. The pairwise distance distributions were balanced out by calculating the ranking of molecular pairs for every compound, resulting in 1213 ranked lists of 1213 pairs each for the FDA set, 1500 ranked lists of 1500 ranked pairs for the EGFR set and 274 ranked lists of 274 pairs for the polymyxin B similarity set.

### Reaction informatics

A reaction SMILES in the form “SMILES1 » SMILES2” (forward reaction) as well as “SMILES2 » SMILES1” (backward reaction) was generated for every selected molecular pair. The forward reaction SMILES was generated to always have the molecule with the lower heavy atom count as a reactant and the molecule with the higher heavy atom count as a product. The reaction SMILES for each drug pair was then encoded using DRFP.^21^ The 20 nearest neighbors (NNs) in the DRFP feature space were extracted and the minimum spanning tree layout calculated using the TMAP package.^29^ The resulting layout was displayed interactively using Faerun.^46^ In addition, the atom-mapping and the corresponding atom-mapping confidence scores were computed for each drug pair reaction SMILES using the published model described in the RXNmapper^22^ GitHub repository https://github.com/rxn4chemistry/rxnmapper.

## Results and discussion

### Datasets and selection of drug pairs

To test our reaction informatics approach to map drug space, we selected 1213 FDA-approved drugs as a representative high diversity set. As examples of a more focused series, we accessed the ChEMBL database^37^ and retrieved 1500 analogs of the small molecule drug afatinib, a kinase inhibitor blocking the endothelial growth factor receptor (EGFR) and used to treat non-small cell lung carcinoma (NSCLC),^47^ as well as 274 analogs of polymyxin B (PMB), an FDA-approved macrocyclic peptide natural product considered as a last resort antibiotic against multidrug-resistant bacteria.^48^

To represent molecular similarities, we considered three types of molecular fingerprints. First, we selected the classical Morgan fingerprint,^38^ also called extended connectivity fingerprint (ECFP),^39^ which is a binary fingerprint encoding the presence of specific atom-centered circular substructures up to a diameter of four (ECFP4) and six (ECFP6) bonds, as well as our recently reported MinHashed fingerprint MHFP6,^40^ which similarly encodes circular substructures up to a diameter of six bonds using shingling and MinHashing to compress information.^49^ These circular substructure fingerprints are particularly efficient in virtual screening benchmarks^40,50^ and off-target prediction tasks.^51,52^ Second, we considered three pharmacophore fingerprints encoding the relative positions of atoms in a molecule and representing molecular shape, namely the RDKit atom-pair fingerprint AP,^41^ our recently reported macromolecule extended atom-pair fingerprint MXFP,^42^ and the MinHashed Atom-pair fingerprint up to a diameter of four bonds MAP4.^43^ Finally, we also included two composition fingerprints, namely MACCS keys^44^ and molecular quantum numbers (MQN),^45^ which encode the presence and number of features present in a molecule.

To identify relevant pairs in each of our three drug sets (FDA, EGFR and PMB), we computed all pairwise distances in each fingerprint as either Jaccard distance *d*_J_ (ECFP4, ECFP6, MHFP6, AP, MAP4, MACCS keys) or Taxicab distance *d*_T_ (MXFP, MQN). For all fingerprints, distance zero indicates highest similarity. For each molecule in each set, we then selected the NN for each of the eight fingerprints, as well as any molecule appearing in at least seven of the eight lists of top-20 nearest neighbors. In addition, we selected all drug pairs having a certain similarity in each fingerprint by applying a maximum Jaccard distance (*d*_J_) threshold (see Methods for details).

**Fig. 2:**
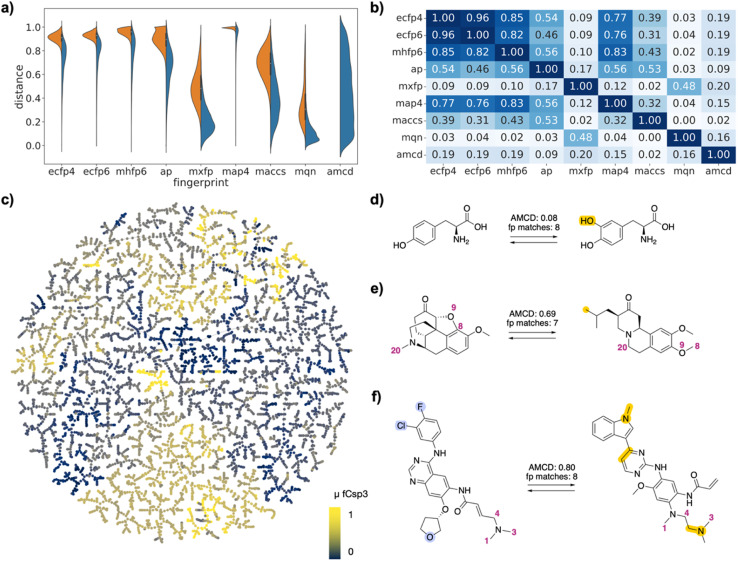
FDA-approved drugs as drug pairs. (a) Violin plot of *d*_J_ values in each of the fingerprints for all pairs (left, orange) or for selected pairs (right, blue), and for atom mapping confidence distance (amcd) of selected pairs (blue, last entry). (b) Heat map of correlation coefficients *r*^2^ between *d*_J_ values of different fingerprints, and between *d*_J_ values and amcd, calculated across all selected pairs. (c) TMAP of DRFP similarities for selected drug pairs. Each point is a different drug pair, color-coded by the fraction of sp^3^ atoms (Fsp^3^). See ESI† and https://tm.gdb.tools/map4/DRFP_FDA/ for additional color codes and for the interactive version of the map. (d) Atom-mapped drug pair l-tyrosine and l-DOPA related by a hydroxylation reaction. (e) Atom-mapped drug pair tetrabenazine and hydrocodone related by an alchemical double cyclization. (f) Atom-mapped drug pair afatinib and osimertinib related by a series of substituent and ring system changes. Atoms highlighted in blue are lost during the forward reaction, while atoms highlighted in yellow are gained. Interesting atom rearrangements as predicted by the RXNMapper are highlighted with their respective atom-mapping number. The full atom-mapping can be found in Fig. S3.†

This selection corresponded to 6406 (0.87%) of the 735 078 possible drug pairs in the FDA set, 8932 (0.79%) of the 1 124 250 possible drug pairs in the EGFR set, and 8464 (22.63%) of the 37 401 possible drug pairs in the PMB set. Each drug was represented in the selected pairs between 1 and 193 times in the FDA approved set, between 1 and 870 times in the EGFR set, and between 4 and 1031 times in the PMB set (Fig. S1†). Compared to the exhaustive list of drug pairs, the selected drug pairs were enriched in high similarity pairs with lower values of Jaccard distance (*d*_J_). They spanned the entire similarity range in each fingerprint, reflecting the fact that the different fingerprints captured different similarity features (Fig. 2a/3a/4a). Distances were correlated between ECFP4, ECFP6, MHFP6, MAP4, which all encode circular substructures around atoms (*r*^2^ ∼ 0.8, Fig. 2b/3b/4b). However, correlations of MAP4 with other circular substructure fingerprints, particularly in the polymyxin B2 set, were generally lower. This can be attributed to its hybrid nature, which encodes both substructures and atom-pairs. Even so, the correlation between MAP4 and circular substructure fingerprints was notably stronger than its correlation with other fingerprint types. AP and MACCS, which both encode atomic features, were weakly correlated with each other and to a lesser extent with circular fingerprints (*r*^2^ ∼ 0.5). Finally, MQN and MXFP distances were partly correlated with each other (*r*^2^ ∼ 0.5) but not with any other fingerprints, probably because both fingerprints are size-dependent and count similar features in molecules.

### DRFP chemical space maps

To gain a closer insight into the pairwise relationships among the selected drug pairs, we represented each pair in the form of a reaction SMILES considering the conversion of one drug into the other. Form the reaction SMILES, we then computed the differential reaction fingerprint (DRFP),^21^ which encodes the circular substructures that occur only in either the reactant or the product. To represent the DRFP chemical space illustrating the similarities between different drug pairs, we then computed a tree-map (TMAP) providing an overview of drug pairs in each of the three datasets, using various color codes to visualize pair properties (Fig. 2c/3c/4c). The TMAP of DRFP similarities organized pairs by structural types, often series of close analogs of a reference drug. Furthermore, in the FDA-approved drug set, different compound families such as amino acids, steroids, β-lactams, catecholamines, benzodiazepines or prostaglandins appeared in different regions of the map. This was visible upon close inspection of the interactive TMAPs and is illustrated here for the FDA drug set with the color FCsp^3^ (Fig. 2c).

**Fig. 3:**
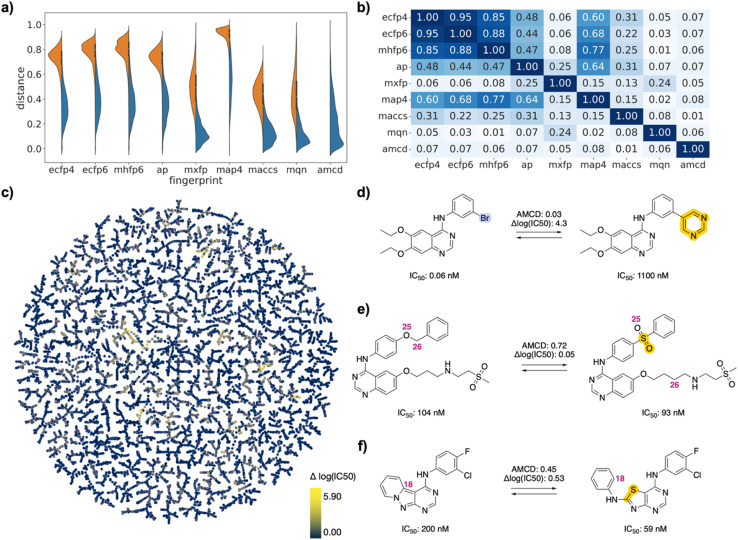
EGFR inhibitor drug pairs. (a) Violin plot of *d*_J_ values in each of the fingerprints for all pairs (left, orange) or for selected pairs (right, blue), and for atom mapping confidence distance (amcd) of selected pairs (blue, last entry). (b) Heat map of correlation coefficients *r*^2^ between *d*_J_ values of different fingerprints, and between *d*_J_ values and amcd, calculated across all selected pairs. (c) TMAP of activity differences. Each point is a different drug pair, color-coded by the activity difference. See ESI† and https://tm.gdb.tools/map4/DRFP_EGFR/for additional color codes and for the interactive version of the map. (d) Atom-mapped drug pair CHEMBL35820 and CHEMBL126974 related by a Suzuki coupling resulting in an activity cliff. (e) Atom-mapped drug pair CHEMBL460732 and CHEMBL14952 related by an alchemical double linker exchange preserving activity (f) Atom-mapped drug pair CHEMBL469997 and CHEMBL181275 related by an alchemical scaffold hopping preserving activity. Atoms highlighted in blue are lost during the forward reaction, while atoms highlighted in yellow are gained. Interesting atom rearrangements as predicted by the RXNMapper are highlighted with their respective atom-mapping number. The full atom-mapping can be found in Fig. S4.†

Interactive browsing of the TMAPs made it very easy to inspect drug pairs with specific properties. For example, with the EGFR set, color-coding by activity differences pointed to the few similar drug pairs representing activity cliffs (Fig. 3c). Inspection of TMAPs was also key to identifying interesting pairs from the point of view of their transformations, as discussed below.

### Atom mapping

To estimate whether paired drugs were interconvertible by a feasible chemical reaction or required a more esoteric transmutation, we subjected the drug pair reaction SMILES to the Transformer-based RXNMapper model,^22^ which returns an atom-to-atom comparison illustrating the structural relationships within pairs, as well as an atom-mapping confidence score. Atom-mapping confidence scores were determined for the forward and backward reactions and converted to atom-mapping confidence distances (amcd), defined here as one minus the confidence score. In most cases the amcd values were similar for forward and backward reactions, however since the difference was sometimes substantial (Fig. S2†), we used the mean amcd of forward and backward reactions for our analysis. The mean amcd value spanned the entire range between low and high distance (last entry, Fig. 2a/3a/4a) except for the PMB set, which mainly contains high confidence distances as the structures are too big for the model to map with high confidence. Further, the amcd was not correlated with any of the selected molecular similarities (last entry, Fig. 2b/3b/4b).

Low amcd values indicated drug pairs related by a simple and usually feasible chemical transformation, usually a functional group change or addition as those found in matched molecular pairs,^31,32^ illustrated in the FDA set for the hydroxylation of l-tyrosine to l-DOPA (Fig. 2d), and in the EGFR set for a Suzuki coupling resulting in a large activity change (Fig. 3d). In the case of the PMB set, low amcd values indicated pairs related by single amino acid exchange often potentially corresponding to a reaction, for example mutation of a glycine to a phenylalanine residue corresponding formally to an α-alkylation of glycine with benzyl bromide (Fig. S6†). This observation suggests that the amcd metric effectively captures chemically intuitive transformations, aligning well with the way chemists predict and perceive such changes in molecules during drug design and development.

On the other hand, high amcd values indicated alchemical transmutations that cannot be realized easily, such as scaffold-hopping changes.^33,34^ Note that the RXNMapper assigned corresponding atoms mostly in a correct manner even for pairs giving high amcd values. For example, tetrabenazine is paired with hydrocodone by seven of the eight molecule fingerprints used for pairing. The transformation features an exotic double-ring formation accompanied by a reshuffling of the 23 atoms (Fig. 2e). A similarly exotic alchemical change relates afatinib with osimertinib, an analog matched by all eight fingerprints used for pairing (Fig. 2f). In the EGFR set, a double linker modification preserving activity relates CHEMBL469997 to CHEMBL181275, whereby the benzyl ether linker is obtained by combining an oxygen atom of the sulfone with a methylene group of the aminobutanol second linker group (Fig. 3e). In another scaffold hopping change between CHEMBL469997 and CHEMBL181275, an aniline substituent is incorporated into the adjacent bicyclic system to form a condensed tricyclic heteroaromatic group, resulting in an interesting activity increase (Fig. 3f).

**Fig. 4:**
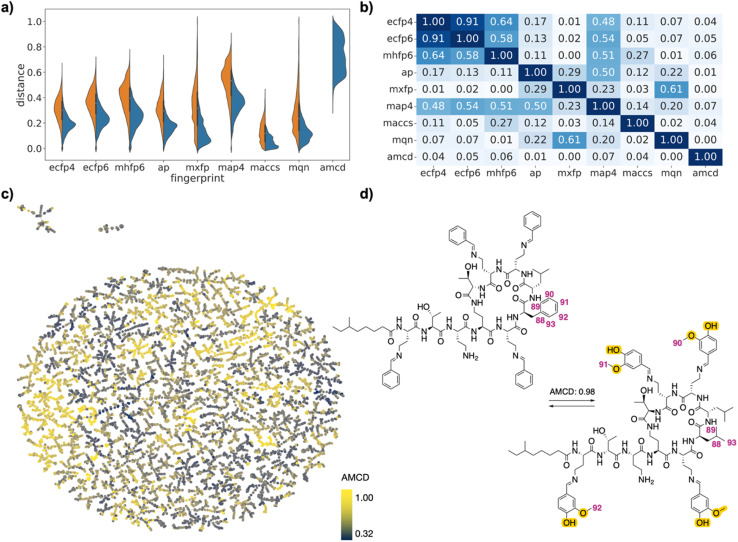
PMB analogs drug pairs. (a) Violin plot of *d*_J_ values in each of the fingerprints for all pairs (left, orange) or for selected pairs (right, blue), and for atom mapping confidence distance (amcd) of selected pairs (blue, last entry). (b) Heat map of correlation coefficients *r*^2^ between *d*_J_ values of different fingerprints, and between *d*_J_ values and amcd, calculated across all selected pairs. (c) TMAP of amcd values. Each point is a different drug pair, color-coded by the amcd value. See ESI† and https://tm.gdb.tools/map4/DRFP_PMB/for additional color codes and for the interactive version of the map. (d) Atom-mapped drug pair CHEMBL1090265 and CHEMBL2372545 related by an imine exchange and a leucine → phenylalanine mutation. Atoms highlighted in blue are lost during the forward reaction, while atoms highlighted in yellow are gained. Interesting atom rearrangements as predicted by the RXNMapper are highlighted with their respective atom-mapping number. The full atom-mapping can be found in Fig. S5.†

In the case of the PMB set, many pairs were generally related by high amcd values, probably because the changes corresponded to multiple amino acid exchanges, which cannot be realized on the complete molecules since each sequence analog requires a separate synthesis. Interestingly, one of the high amcd changes corresponds to a simple exchange of four aromatic aldehyde imines attached to the four diaminobutanoic acid residues, a reaction which would seem to be feasible (Fig. 4d). This imine exchange is however accompanied by a mutation of a leucine residue to a phenylalanine.

Taken together, the analysis of the TMAP of similar drug pairs guided by DRFP similarity and amcd values allowed a rapid insight into multiple interesting comparisons between molecules in each of the three sets analyzed. Further examples of interesting pairs in the FDA approved set are provided in the ESI† (Fig. S7†).

## Conclusion

In summary, we have shown that borrowing tools from reaction informatics provides an opportunity to map multiple similarity relationships between molecules simultaneously and gain insights into interesting drug pairs that are otherwise difficult to identify. Specifically, we used DRFP to map the chemical space of multiple drug pairs selected as being similar according to eight different molecular fingerprints simultaneously in the form of TMAPs. We then used RXNMapper to visualize the structural changes between drugs and identify pairs of drugs related by feasible chemical transformation from pairs related by alchemical changes corresponding to multiple and complex structural rearrangements. These tools should generally be applicable to analyze drug sets from multiple angles in the context of drug discovery. One specific case could be the analysis of analog series obtained from generative models^53,54^ to help identify feasible transformations or single out scaffold hopping changes.

## Code availability

The source codes and datasets used for this study are available at https://github.com/reymond-group/alchemical_pairs.

## Author contributions

MO designed and realized the project and wrote the paper. DP provided support for the DRFP implementation and wrote the paper. PS provided support for the RXNmapper implementation and wrote the paper. JLR designed and supervised the project and wrote the paper. All authors read and approved the final manuscript.

## Conflicts of interest

There are no conflicts to declare.

## Supplementary Material

DD-002-D3DD00039G-s001
