# Supplementary material for: Alchemical analysis of FDA approved drugs
Source: Digit Discov. 2023 Aug 30;2(5):1289–96. doi: 10.1039/d3dd00039g (PMC10561545; doi:10.1039/d3dd00039g)
Supplement: DD-002-D3DD00039G-s001 [file DD-002-D3DD00039G-s001.pdf]

Electronic Supplementary Material (ESI) for Digital Discovery.
This journal is © The Royal Society of Chemistry 2023

Supplementary Information for: Alchemical Analysis of FDA Approved Drugs

Markus Orsi,[a] Daniel Probst,[b] Philippe Schwaller[b] and Jean-Louis Reymond[a]*

[a)] Department of Chemistry, Biochemistry and Pharmaceutical Sciences, University of Bern, Freiestrasse 3, 3012 Bern, Switzerland, [b)]Ecole Polytechnique Fédérale de Lausanne, 1015 Lausanne, Switzerland

e-mail: *jean-louis.reymond@unibe.ch*

Table of Contents

1. Supplementary figures

Figure S1 2

Figure S2 3

Figure S3 4

Figure S4 5

Figure S5 6

Figure S6 6

Figure S7 7

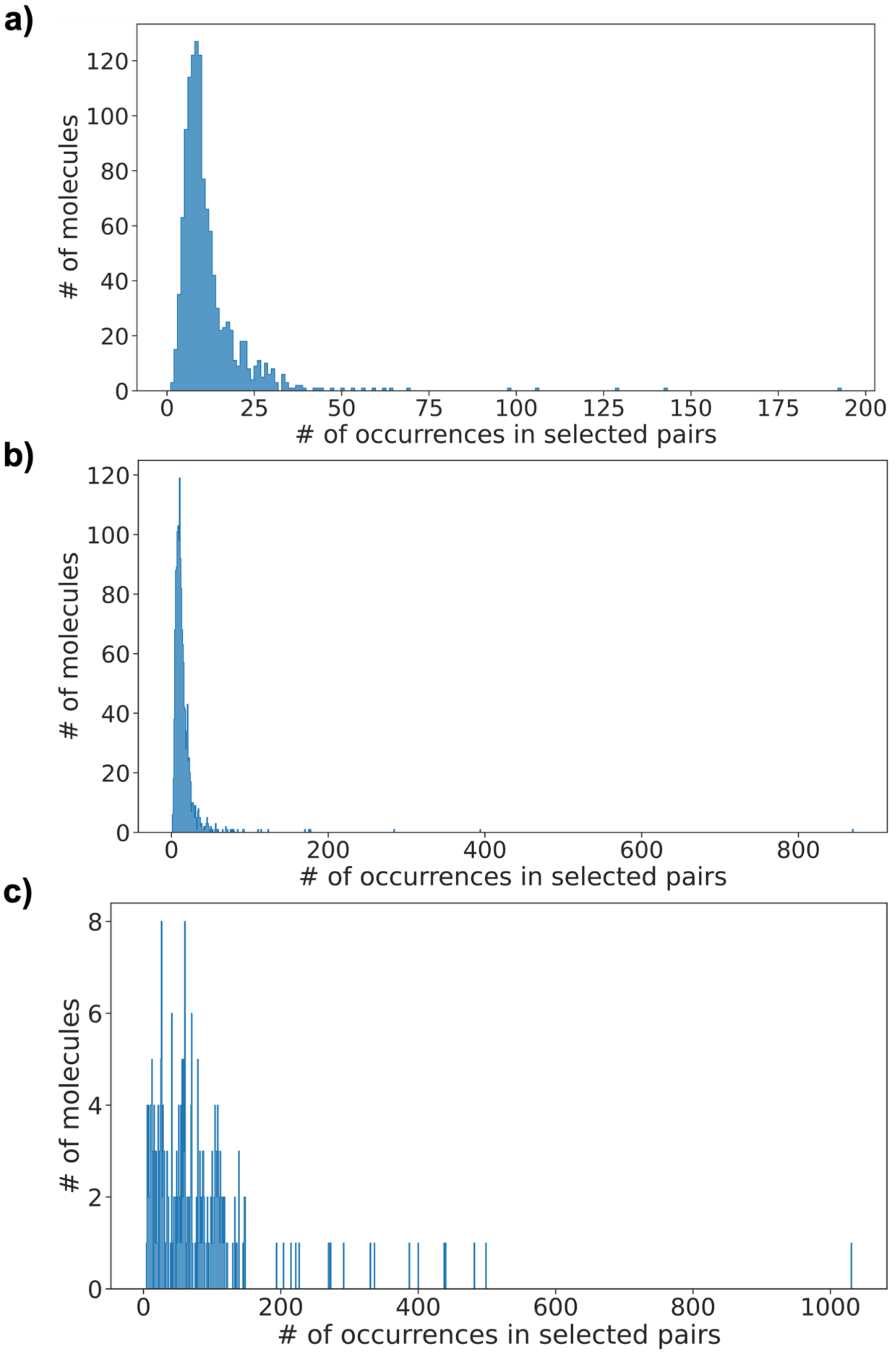

Figure S1. Count of molecules by number of occurrences in selected pairs for the a) FDA, b) EGFR and c) PMB set. In all sets, most of the molecules appear sporadically in the selected pairs. Only a limited number of compounds appears more often.

a)

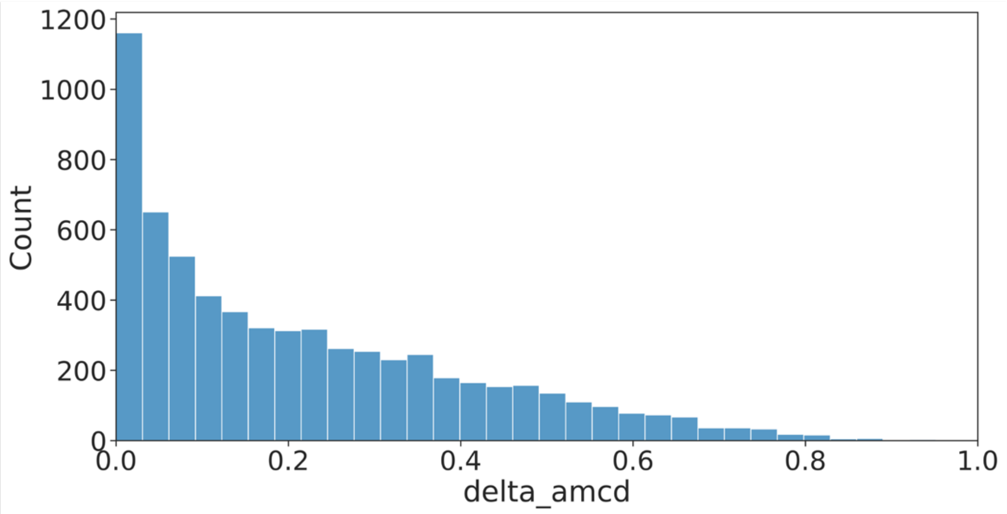

b)

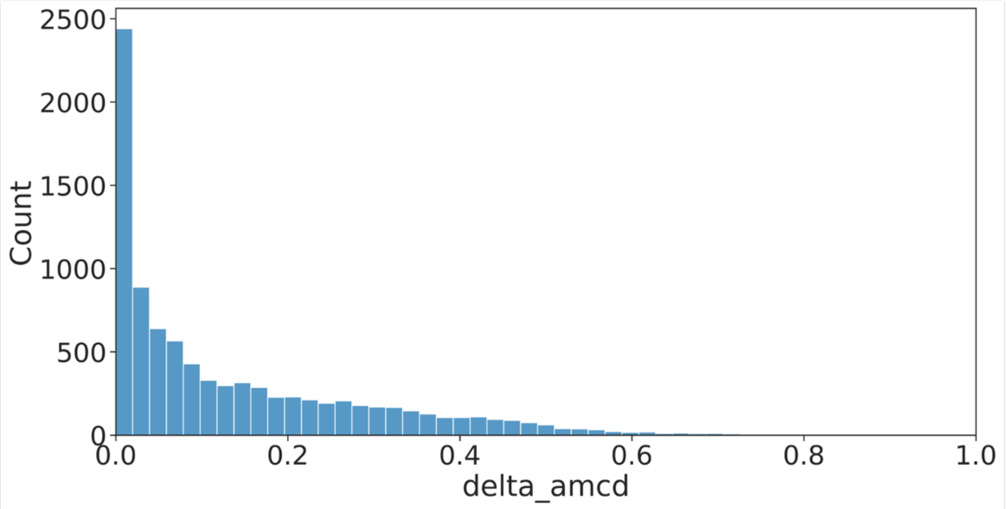

c)

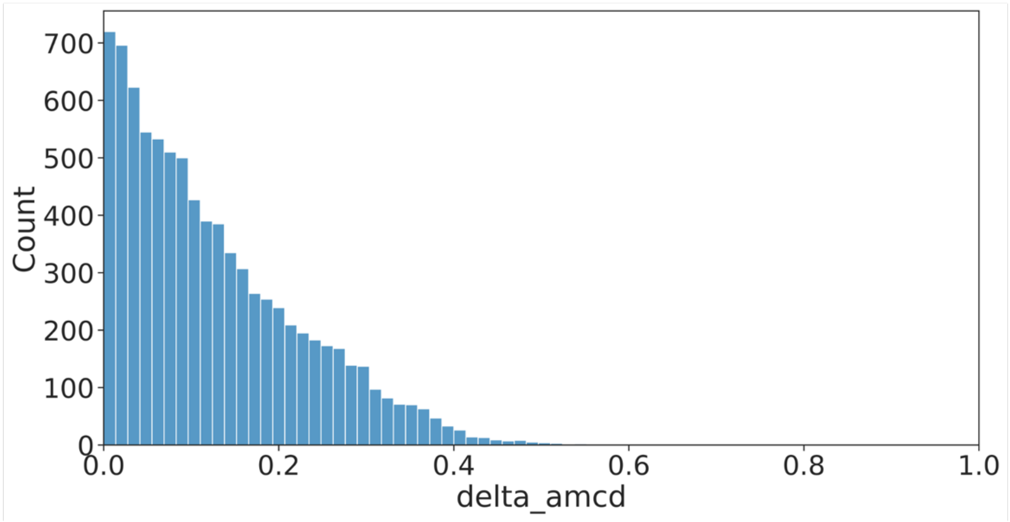

Figure S2. Count of molecular pairs by difference in atom-mapping confidence score between the forward and backward reactions in selected pairs for the a) FDA, b) EGFR and c) PMB set.

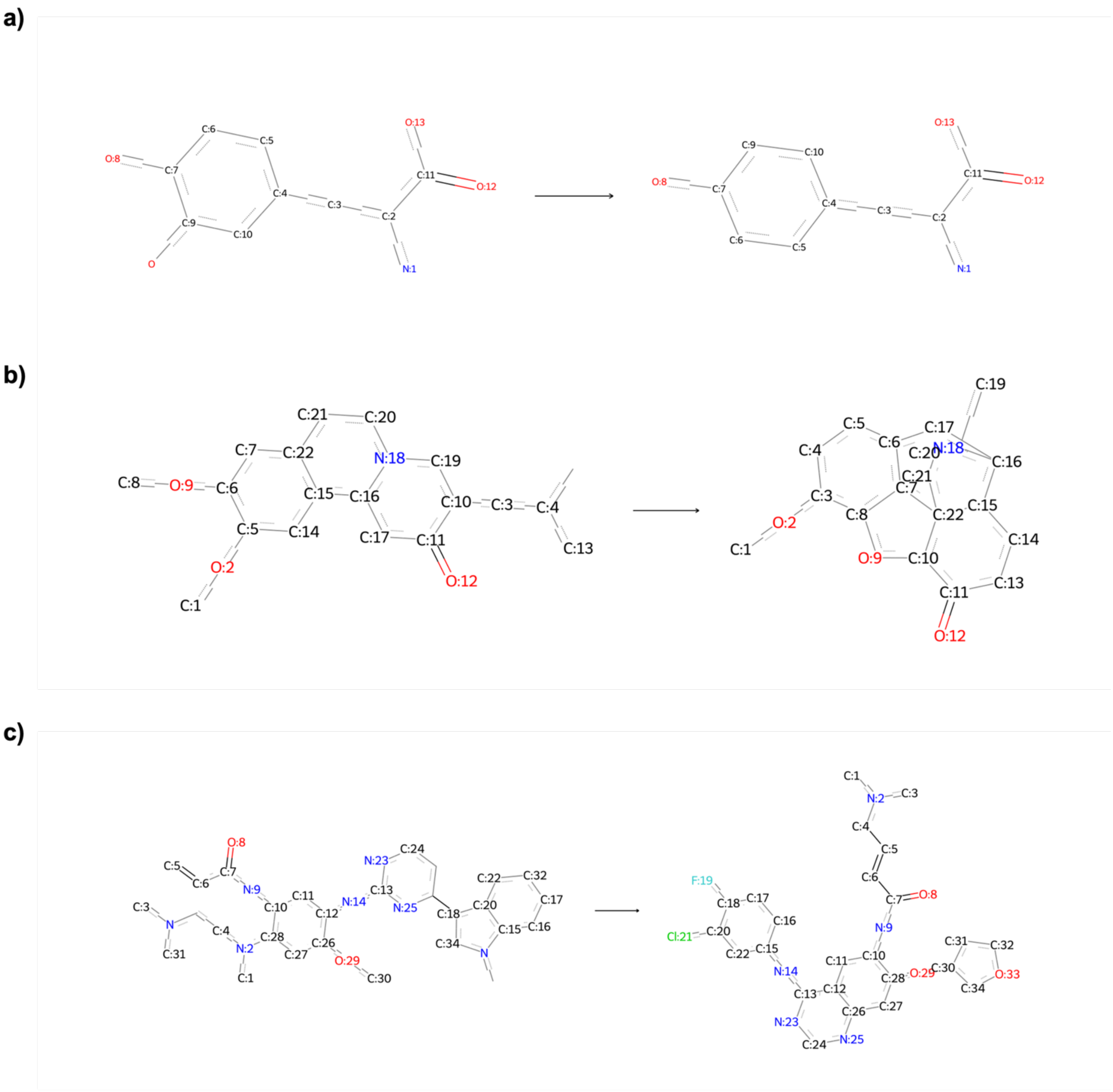

Figure S3. Full atom mapping of the examples selected from the FDA set. The shown atom-mapping is the one of the backwards reactions.

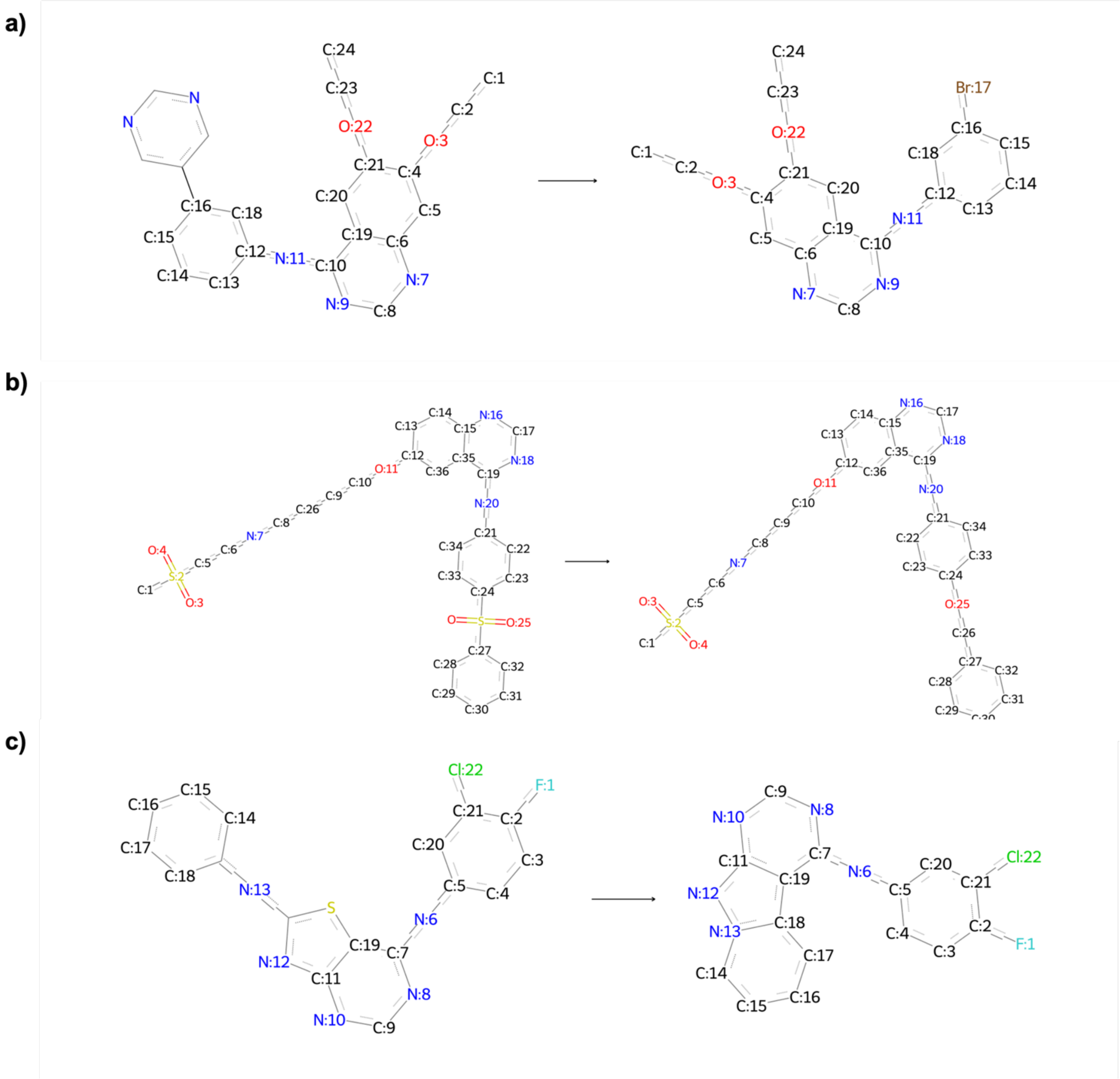

Figure S4. Full atom mapping of the examples selected from the EGFR set. The shown atom-mapping is the one of the backwards reactions.

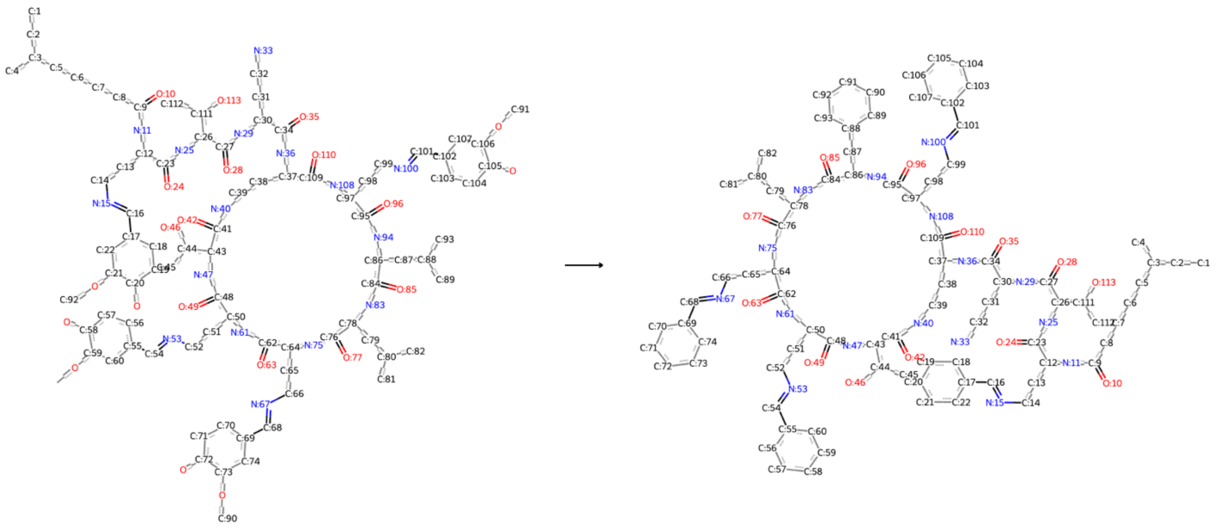

Figure S5. Full atom mapping of the example selected from the PMB set. The shown atom-mapping is the one of the backwards reaction.

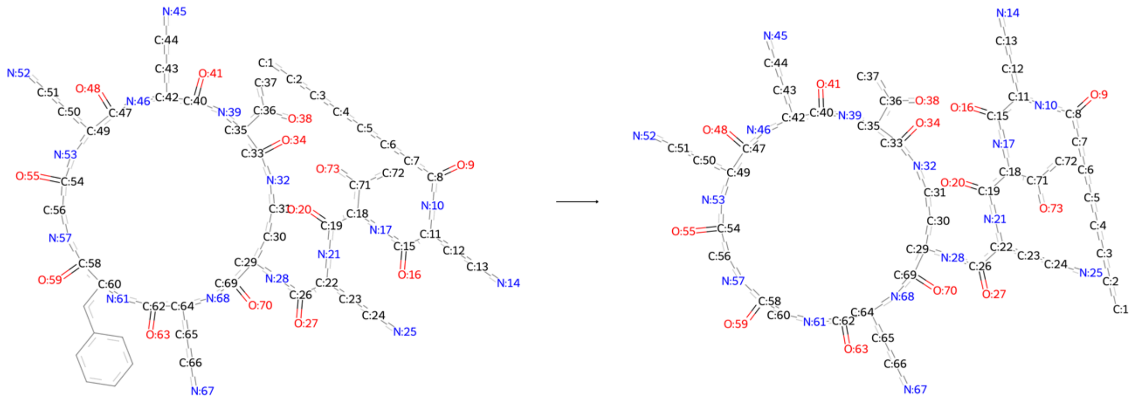

Figure S6: Full atom-mapping of mutation of a glycine to a phenylalanine residue (amcd: 0.32), corresponding to a feasible α-alkylation reaction of glycine with benzyl bromide.

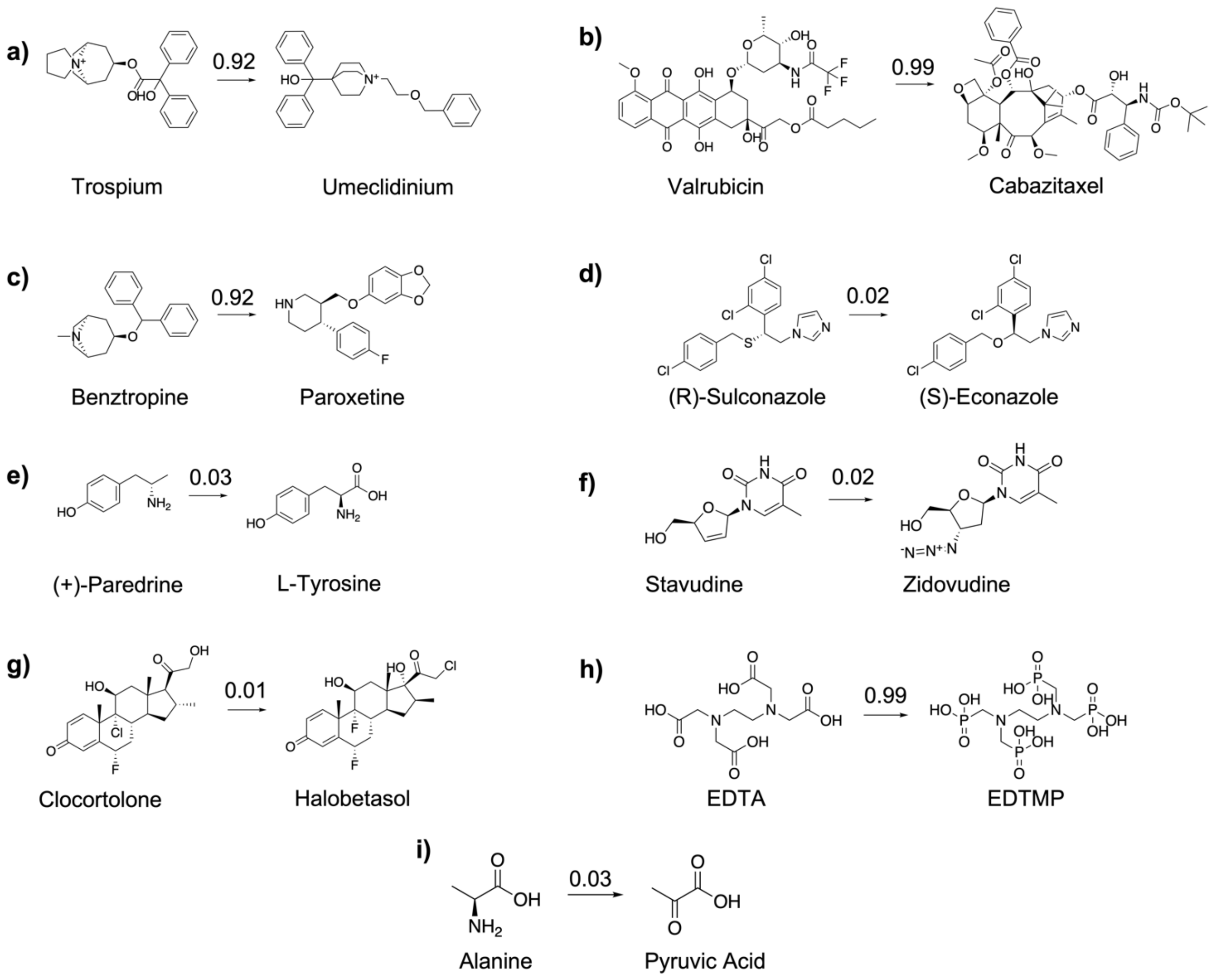

Figure S7: Additional interesting pairs selected from the drug pairs in the FDA-approved subset and the determined atom-mapping confidence distance of the reaction. a) Trospium and Umeclidinium, two anticholinergic drugs acting on the muscarinic receptor. The structures contain common elements, such as the diphenylmethanol and tropane-like moieties, which are completely rearranged between the two structures. b) Valrubicin and Cabazitaxel, two anticancer drugs acting on topoisomerase II and tubulin stabilization respectively. Although the two compounds act on different targets, these targets are part of the same pathway and their inhibition leads to cell death. c) Benztropine and Paroxetine, two unrelated drugs acting on serotonin uptake inhibition. d) (R)-Sulconazole and (S)-Econazole, two imidazole antifungals differing from each other by a single atom mutation from S to O. e) (+)-Paredrine and L-Tyrosine, two closely related structures separated by an alchemical condensation of a carboxylic acid to a methyl and stereo-inversion. f) Stavudine and Zidovudine, two HIV reverse transcriptase inhibitors separated by an azidation. g) Clocortolone and Halobetasol, two steroid drugs used for the treatment of inflammatory and itching skin diseases. h) EDTA and EDTMP, both highly related chelating agents. i) Alanine and Pyruvic Acid, two highly related compounds separated by a N to =O mutation.
